# Field and Hospital Stretcher

**Published:** 1863-10

**Authors:** 


Field and Hospital Stretcher.—Dr. Frank H. Hamilton has just perfected an admirable invention of a field and hospital stretcher, which is at the same time as comfortable a bed as any man, sick or well, could desire. It is the surgeon's and nurse's long-sought desideratum, and is as well adapted for use in private families as in the army. The chief merits of it are compactness and facility for moving or changing the position of the patient without touching his body or lifting him by the limbs, a process that is dangerous and often impossible with wounded men on the usual plans of support. It will be a positive preventive of the terrible bed sores from which the soldier often suffers more than from broken limbs. Two or three hundred of Dr. Hamilton's stretchers can be carried in a single wagon, and it does not take three minutes to put them in trim. The greatest advantage will be gained by them in removing the wounded from a field. The canvass can be drawn under a body in strips so gently as not to provoke or increase the bleeding of wounds, and being buttoned to the side-pieces and stretched by a simple screw, the bed can be carried off, or supported on stones, as comfortably to the patient as on a regular four-poster. The model will be presented to the Surgeon-General in a few days.—*Med. and Surgical Reporter.*

